# Assessing the Impact of Flavophospholipol and Virginiamycin Supplementation on the Broiler Microbiota: a Prospective Controlled Intervention Study

**DOI:** 10.1128/msystems.00381-21

**Published:** 2021-08-31

**Authors:** Mohamed Mysara, Matilda Berkell, Basil Britto Xavier, Sarah De Backer, Christine Lammens, Veerle Hautekiet, Spas Petkov, Herman Goossens, Samir Kumar-Singh, Surbhi Malhotra-Kumar

**Affiliations:** a Lab of Medical Microbiology, Faculty of Medicine and Health Sciences, Vaccine & Infectious Disease Institute, University of Antwerp, Antwerp, Belgium; b Microbiology Unit, Interdisciplinary Biosciences, Belgian Nuclear Research Centre, SCK•CEN, Mol, Belgium; c Molecular Pathology group, Cell Biology & Histology, Faculty of Medicine and Health Sciences, University of Antwerp, Antwerp, Belgium; d Huvepharma, Antwerp, Belgium; e Huvepharma, Sofia, Bulgaria; University of California San Diego

**Keywords:** flavomycin, flavophospholipol, virginiamycin, broiler chicken, microbiota, 16S rRNA sequencing

## Abstract

The antibiotic growth promoters (AGPs) flavophospholipol and virginiamycin have been widely used for decades in food animal production. AGP activity is believed to be partly modulated by gut microbial composition although exact AGP-induced changes remain unclear. In a controlled intervention study, we studied the effect of flavophospholipol and virginiamycin on the broiler chicken ileal microbiota spanning from birth to 39 days. Using 16S rRNA gene profiling and prediction of metabolic activity, we show that both AGPs result in dynamic microbial shifts that potentially increase anti-inflammatory mechanisms and bioavailability of several essential nutrients by decreasing degradation (flavophospholipol) or increasing biosynthesis (virginiamycin). Further, virginiamycin-supplemented broilers showed increased colonization with potentially pathogenic bacteria, Clostridium perfringens, Campylobacter, and Escherichia/*Shigella* spp. Overall, we show that both AGPs induce microbial changes potentially beneficial for growth. However, the increase in (foodborne) pathogens shown here with virginiamycin use could impact not only broiler mortality but also human health.

**IMPORTANCE** Antibiotic growth promoters (AGPs) are commonly used within poultry farming to increase muscle growth. Microbial composition in the gut is known to be influenced by AGP use although exact AGP-induced changes remain unclear. Utilizing 16S rRNA gene profiling, this study provides a first head-to-head comparison of the effect of the two most commonly used AGPs, flavophospholipol and virginiamycin, on the broiler chicken ileum microbiota over time. We found that supplementation with both AGPs altered ileal microbial composition, thereby increasing potential bioavailability of essential nutrients and weight gain. Flavophospholipol showed a slight benefit over virginiamycin as the latter resulted in more extensive microbial perturbations including increased colonization by enteropathogens, which could impact broiler mortality.

## INTRODUCTION

Antibiotic growth promoters (AGPs) have been widely used in broiler chickens to improve growth performance in the farming of food animals for over 60 years, including flavophospholipol and virginiamycin (1, 2). Flavophospholipol (synonyms: bambermycin, moenomycin) is a phosphoglycolipid antimicrobial produced by *Streptomyces* spp. that inhibits cell wall synthesis of primarily Gram-positive bacteria (3, 4). Virginiamycin belongs to the streptogramin class and consists of two active components that inhibit protein synthesis by binding to 23S rRNA of the 50S ribosomal subunit (5). Although a beneficial effect in promoting muscle growth has been reported for both, the underlying mechanisms by which APGs promote growth are not fully understood (6, 7). It is suggested that the promoting activity of AGPs is in part driven by a modulation of the microbial composition in the gut, including, but not limited to, suppression of pathogenic bacteria (8, 9). For instance, flavophospholipol is known to suppress certain microorganisms (e.g., Staphylococcus xylosus and Enterococcus faecalis) and contribute to an improved equilibrium of the gut microflora by providing a barrier that prevents colonization by pathogenic bacteria, which is associated with improved weight gain and feed conversion (10). Additionally, reduced shedding of pathogenic bacteria, such as Salmonella spp., has been reported for flavophospholipol in pigs, calves, and chickens (11, 12). Similarly, virginiamycin has also been reported to induce gut microbial shifts in broiler chickens (13, 14). This microbial shift, mainly reported in the ileum, was characterized by an overrepresentation of species within the two genera *Corynebacterium* and *Propionibacterium* (13, 14). The latter is capable of producing propionate, a short-chain fatty acid (SCFA) that has immune modulatory capacity, and of binding aflatoxin B1, a major feed contaminant in the poultry industry, to reduce its uptake in the intestine (13). However, other studies have shown virginiamycin use to be strongly associated with increased colonization by opportunistic pathogens such as Clostridium perfringens, linked to higher mortality rates (15, 16).

The broiler chicken gut microbiome consists of a complex mixture of bacteria, archaea, viruses, and fungi that play a vital role in nutrient absorption, immunity, physiological development, and protection from pathogens (17). Previous studies have indicated that different functions are contained within each part of the digestive system as a result of differences in microbial communities harbored in each organ (18, 19). The small intestine microbiota is well delineated, in particular the ileum microbiota, which is dominated by lactobacilli, enterococci, and *S. xylosus* (18, 20). Several factors, such as changes in diet, housing, temperature, and antibiotics, have been shown to affect microbial composition (21). Furthermore, AGPs have been shown to result in morphological changes in the gut, such as a distinct reduction in gut weight and wall thickness, which could contribute to an increased nutritional uptake (22, 23).

Concerns related to the impact of AGP use in poultry farming on the spread of antimicrobial resistance have been raised (24, 25), and as a precautionary measure the usage of virginiamycin and flavophospholipol was banned in 1999 and 2006, respectively, in the European Union (EU) (26), but they are still frequently used as AGPs outside the EU. Previous studies have investigated the effect of individual AGPs on the broiler chicken microbiome, but interventional studies comparing the effect of multiple AGPs in a controlled setting are rare (6). Moreover, broiler chickens live up to approximately 6 weeks before they are slaughtered, while previous studies have reported microbial changes at different broiler ages that could influence outcome and prevent interstudy comparisons. Here, we conducted a prospective controlled intervention study where newborn broiler chickens were followed from the time of hatching up to 6 weeks to investigate the effects of flavophospholipol or virginiamycin use on the ileum microbiota by utilizing 16S rRNA gene profiling. We here describe overlapping but also discrete changes in dominant bacterial taxa within the gut microbiota as a result of flavophospholipol or virginiamycin supplementation at distinct ages.

## RESULTS

### The diversity and composition of the broiler chicken ileal microbiota evolve with age.

**FIG 1:**
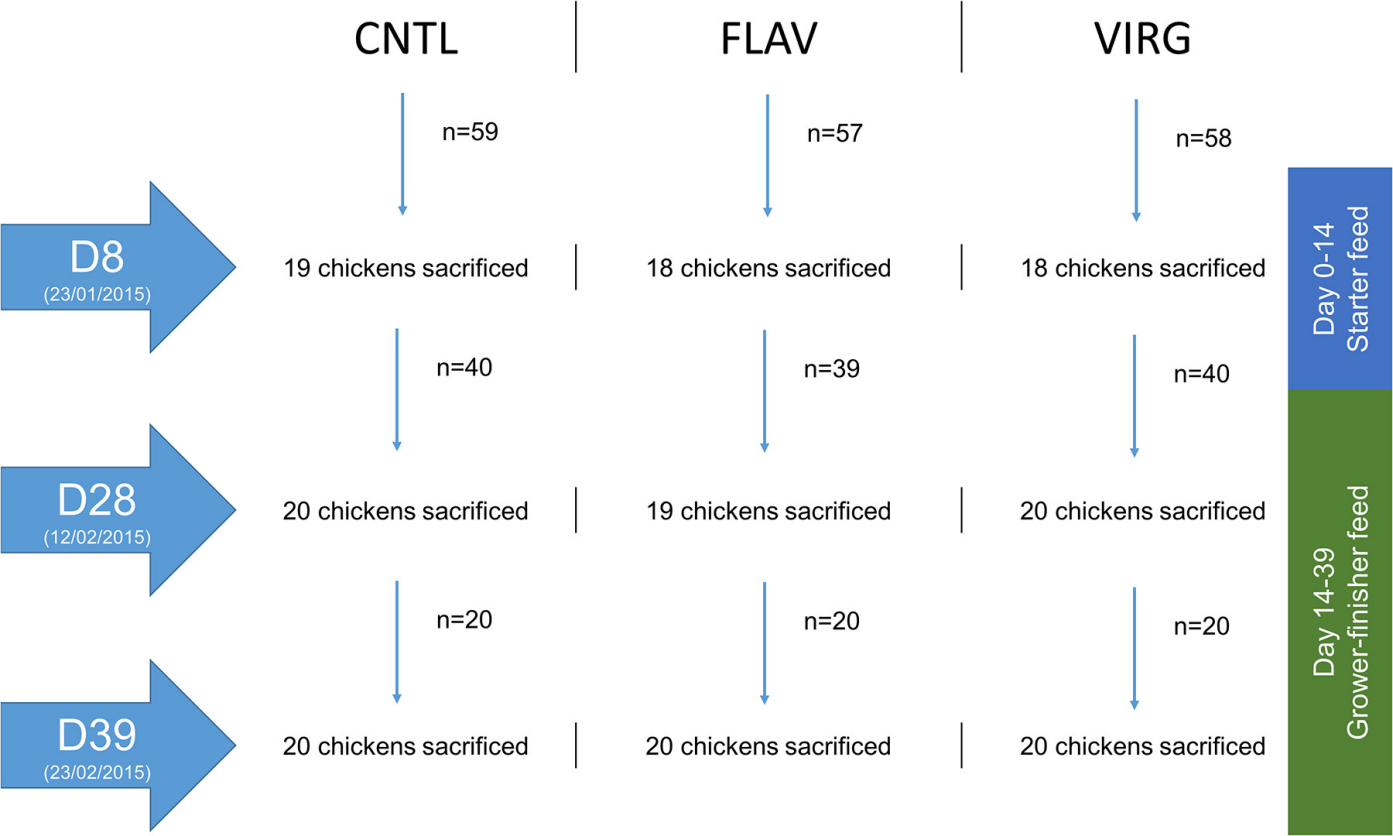
Design of the controlled intervention study investigating the impact of flavophospholipol (FLAV, 15 ppm) and virginiamycin (VIRG, 20 ppm) on the ileum microbiota in broiler chickens. In total, 60 chickens were included in each treatment arm of the study, and 20 each were sacrificed at three distinct time points: after 8 days (D8), 28 days (D28), and 39 days (D39). One chicken died in the control group prior to D8, one in the flavophospholipol-supplemented group prior to D28, and two in the virginiamycin-supplemented group prior to D8. An additional two samples were lost in the flavophospholipol-supplemented group at D8 due to failure of collection or insufficient sample material. Ileal and cecal content was collected at each time point for further analysis using 16S rRNA gene profiling.

A controlled intervention study with three arms was conducted on 180 male broiler chickens during a 6-week period to assess the impact of flavophospholipol and virginiamycin on the ileal and cecal microbiota (Fig. 1). Sixty chickens randomly divided over 10 pens were included in each arm, where they received either 15 ppm flavophospholipol, 20 ppm virginiamycin, or no AGP supplementation. Twenty chickens were sacrificed in each arm, and ileal and cecal contents were collected at day 8 (D8), 28 (D28), and 39 (D39) to assess longitudinal microbial development. One chicken died in the control group prior to D8, one in the flavophospholipol-supplemented group prior to D28, and two in the virginiamycin-supplemented group prior to D8. An additional two samples were lost in the flavophospholipol-supplemented group at D8 due to failure of collection or insufficient sample material. A pilot analysis of ileal and cecal samples collected at different time points in each arm revealed a more profound impact on the ileal microbiota (data not shown). As a result, only analyses concerning the ileal microbial impact are described in this study.

**FIG 2:**
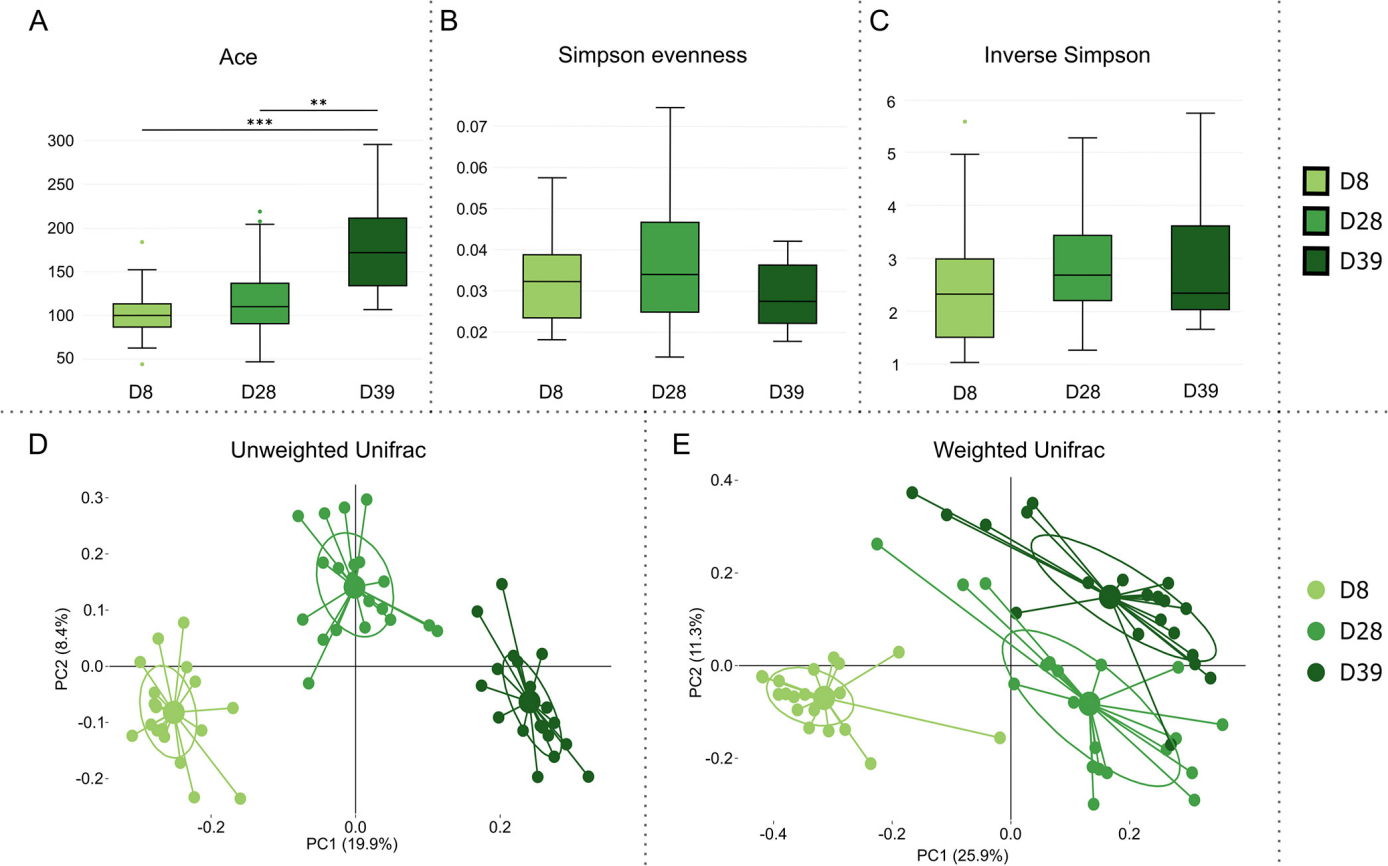
Microbial diversity and composition in ileum of untreated broiler chickens evolve with age. Microbial diversities and compositions were compared at three time points in this study, at days 8 (D8, light green), 28 (D28, medium green), and 39 (D39, dark green). Alpha diversity described by the Ace (A), Simpson evenness (B), and inverse Simpson (C) indices indicates that the overall diversity increases with age. This increase is largely attributed to increased species richness (Ace), whereas the evenness (Simpson evenness) remains stable over time. Differences in microbial composition were demonstrated using principal-coordinate analysis (PCoA) of unweighted (D) and weighted (E) UniFrac beta diversity, revealing that the ileum microbiota changes vastly over time. For detailed information on distinct taxa driving microbial differences reflected in the PCoA, see Table S2 in the supplemental material.

To investigate the evolution of the ileum microbiota over time, alpha and beta diversities were compared at three different time points, D8, D28, and D39, in broiler chickens that did not receive any AGP supplementation. The richness was found to be higher at D39 than at D8 and D28; however, no difference was observed in evenness or overall alpha diversity (Fig. 2A to C). Multidimensional scaling analysis further revealed that the microbial composition changed drastically over time (*P* < 0.001) (Fig. 2D and E; see also Tables S1 and S2 in the supplemental material). In particular, samples from D8 were distinct from latter time points when described by both weighted and unweighted UniFrac principal-coordinate analysis (PCoA) (Fig. 2D and E).

10.1128/mSystems.00381-21.4 TABLE S1 AMOVA and ANOSIM analysis of the control group split over day 8, 28, or 39 (D8, D28, and D39, respectively). Download Table S1, XLSX file, 0.01 MB. Copyright © 2021 Mysara et al. 2021 Mysara et al. https://creativecommons.org/licenses/by/4.0/ This content is distributed under the terms of the Creative Commons Attribution 4.0 International license.

10.1128/mSystems.00381-21.5 TABLE S2 Identification of driving taxa in principal-coordinate analysis (PCoA) of weighted and unweighted UniFrac distances. Specific OTUs associated with individual coordinates/axes in conducted principal-coordinate analysis (PCoA) was determined with an absolute correlation value of >0.7 and a *P* value of <0.05. Download Table S2, XLSX file, 0.01 MB. Copyright © 2021 Mysara et al. 2021 Mysara et al. https://creativecommons.org/licenses/by/4.0/ This content is distributed under the terms of the Creative Commons Attribution 4.0 International license.

Operational taxonomic units (OTUs) that changed distinctly in abundance over time were further classified into distinct types with single-nucleotide resolution using oligotyping (Table S3). The microbial community at D8 was dominated by one OTU classified as *Enterococcus* spp. (closest to E. hirae*/*E. villorum*/*E. ratti*/*E. faecium*/*E. durans) that comprised more than 50% of the average overall abundance (Table 1). Members of the lactobacilli were found to be the second most abundant community (including species within the *Lactobacillus*, *Limosilactobacillus*, and *Ligilactobacillus* genera) wherein several species were detected and classified into several distinct OTUs. Of these, Lactobacillus johnsonii (Otu2) was predominant and formed 19% of the overall microbial community, followed by Lactobacillus crispatus/Lactobacillus gallinarum (Otu5). Additionally, Limosilactobacillus reuteri (Otu8, formerly known as Lactobacillus reuteri) and Ligilactobacillus salivarius (Otu10, formerly known as Lactobacillus salivarius) corresponded to 5% of the overall abundance together with L. crispatus*/L. gallinarum* (Fig. S1). The third most abundant genus was Streptococcus, representing 13% of the chicken’s early microbial community, dominated by one OTU closest to S. pasteurianus*/*S. infantarius*/*S. alactolyticus/S. macedonicus.

**TABLE 1:** Distinctly altered OTUs (*P* < 0.05) in the ileal broiler microbiota in the control group at days 8, 28, and 39 identified by linear discriminant analysis effect size (LDA ≥ 3.0) and Metastats^*a*^

Genus	OTU	Avg abundance (%)			Metastats, *P* value			LEfSe		
		D8	D28	D39	CNTL, D8 vs D28	CNTL, D8 vs D39	CNTL, D28 vs D39	Class	LDA	*P* value
*Enterococcus*	Otu1	54.8	0.5	0.7	0.001	0.001	0.359	D8	5.4	0
*Lactobacillus*	Otu2	19.7	46.1	6.1	0.001	0.001	0.001	D28	5.2	0
Staphylococcus	Otu4	0.1	8.3	12.7	0.001	0.001	0.336	D39	4.7	0
*Clostridiales*	Otu3	0	1.7	0.4	0.001	0.001	0.08	D28	4	0
*Lactobacillus*	Otu5	0.3	15.4	51.2	0.001	0.001	0.001	D39	5.4	0
*Romboutsia*	Otu6	0	0.9	0.9	0.122	0.001	0.976	D28	4	0
Streptococcus	Otu7	13.4	1	0.7	0.001	0.001	0.494	D8	4.7	0
*Limosilactobacillus*	Otu8	1.8	15.4	14.4	0.001	0.001	0.783	D28	4.9	0
*Lachnospiraceae*	Otu9	2.1	1.8	0.2	0.825	0.001	0.019	D8	4.3	0
*Ligilactobacillus*	Otu10	2	0.1	0.5	0.001	0.137	0.555	D8	3.9	0
*Lachnospiraceae*	Otu810	0.2	0	0	0.001	0.001	0.027	D8	3.3	0
*Corynebacterium*	Otu13	0	0.7	3.1	0.001	0.001	0.005	D39	4.1	0
*Blautia*	Otu15	1	0	0	0.001	0.001	0.064	D8	3.7	0
*Brachybacterium*	Otu17	0	0.3	1.6	0.001	0.001	0.013	D39	3.8	0
*Lactococcus*	Otu16	0	0.8	1.1	0.001	0.001	0.451	D39	3.7	0
*Clostridium sensu stricto*	Otu21	0	0	1.5	0.449	0.001	0.001	D39	3.8	0
Escherichia/*Shigella*	Otu32	0.6	0.1	0	0.007	0.001	0.407	D8	3.7	0
*Lachnospiraceae*	Otu36	0.2	0.1	0	0.405	0.005	0.003	D8	3.2	0.002
*Bifidobacterium*	Otu40	0.2	0	0	0.001	0.007	0.2	D8	3.4	0
Streptococcus	Otu50	0	0.5	0.1	0.001	0.001	0.006	D28	3.4	0
*Brevibacterium*	Otu55	0	0.1	0.4	0.071	0.001	0.025	D39	3.4	0
*Lachnospiraceae*	Otu909	0.2	0.1	0	0.199	0.007	0.191	D8	3.2	0.007
*Anaerostipes*	Otu59	0.2	0	0	0.178	0.13	0.692	D8	3.2	0.011
*Limosilactobacillus*	Otu63	0	0.7	0.2	0.001	0.001	0.213	D28	3.4	0
Staphylococcus	Otu66	0	0	0.4	0.001	0.001	0.002	D39	3.2	0
*Blautia*	Otu1041	0.2	0	0	0.086	0.005	0.026	D8	3.4	0.028
*Limosilactobacillus*	Otu91	0	0.2	0.2	0.001	0.001	0.517	D39	3.1	0
*Rhodopseudomonas*	Otu94	0	0	0.2	0.09	0.001	0.001	D39	3	0
*Ligilactobacillus*	Otu109	0	0.4	0.3	0.001	0.001	0.527	D28	3.2	0

a Abbreviations: CNTL, control; D8, day 8; D28, day 28; D39, day 39; LDA, linear discriminant analysis; LEfSe, LDA effect size.

10.1128/mSystems.00381-21.1 FIG S1 Stacked bar plots of the taxonomic composition of the most abundant OTUs, grouped by day of sacrifice (D8, D28, or D39). Download FIG S1, DOCX file, 0.3 MB. Copyright © 2021 Mysara et al. 2021 Mysara et al. https://creativecommons.org/licenses/by/4.0/ This content is distributed under the terms of the Creative Commons Attribution 4.0 International license.

10.1128/mSystems.00381-21.6 TABLE S3 Classification of OTUs of interest using oligotyping. OTUs of interest identified to be distinctly associated with control, flavophospholipol, or virginiamycin broilers at day 8, 28, or 39 were classified into distinct types using oligotyping. Taxonomical classification of each identified type was assigned using NCBI nucleotide BLAST. Only hits with a percent identity of ≥97.0% were considered valid. Download Table S3, XLSX file, 0.01 MB. Copyright © 2021 Mysara et al. 2021 Mysara et al. https://creativecommons.org/licenses/by/4.0/ This content is distributed under the terms of the Creative Commons Attribution 4.0 International license.

The microbial composition underwent a shift during growth (D28 and D39), with a natural reduction in abundance of *Enterococcus* and Streptococcus spp. (*P* = 0.001) and a concomitant increase in lactate-producing bacteria at later time points. These time points were dominated by lactobacilli in particular with an overall relative abundance of approximately 75% at both D28 and D39. Yet, there was a drastic difference in composition among the *Lactobacillus* spp. between D28 and D39. In particular, L. johnsonii was more abundant at D28 (*P* < 0.001) and L. crispatus/*L. gallinarum* at D39 (*P* < 0.001). Additionally, the relative abundance of Staphylococcus xylosus (Otu4) also increased (*P* < 0.001) with age and reached approximately 10% at both D28 and D39, and a similar trend was observed for *Corynebacterium* (Otu13, Fig. S1).

Predictive metabolomic profiling of these microbial compositions identified several pathways to be distinctly represented at D8 in the control group. These included amino acid biosynthesis of glycogen, starch, and lactate and those linked to degradation of lactose and sugar. At the later time points, peptidoglycan and amino acid biosynthesis, fatty acid degradation, and several fatty acid and alcohol fermentation processes were found to be more active (Table S4).

10.1128/mSystems.00381-21.7 TABLE S4 PICRUSt analysis showing distinctly altered pathways in the ileal broiler microbiota in the control (CNTL) group at days 8, 28, and 39 (D8, D28, and D39, respectively). Download Table S4, XLSX file, 0.04 MB. Copyright © 2021 Mysara et al. 2021 Mysara et al. https://creativecommons.org/licenses/by/4.0/ This content is distributed under the terms of the Creative Commons Attribution 4.0 International license.

### Virginiamycin, but not flavophospholipol, supplementation decreases microbial diversity, increases richness, and alters community composition.

**FIG 3:**
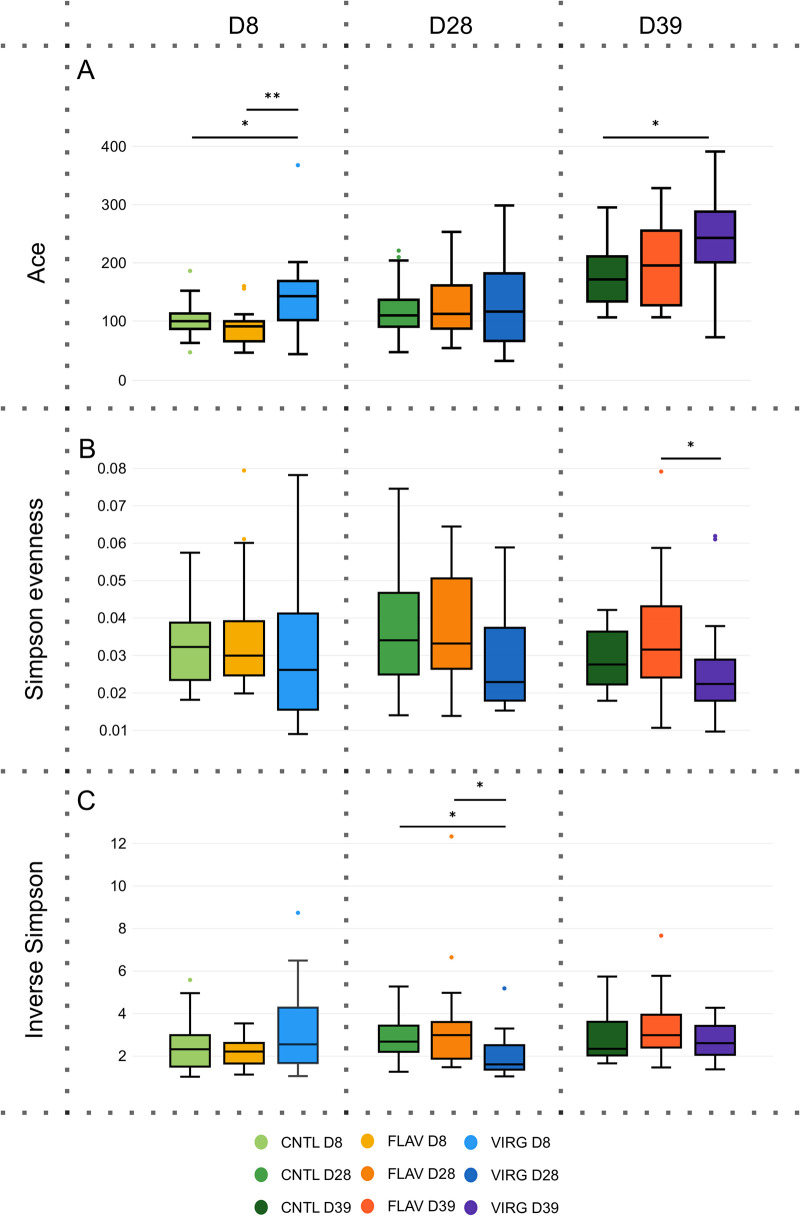
The ileal microbiota of the broiler chicken supplemented with virginiamycin (VIRG) and flavophospholipol (FLAV) evolves similarly to the control group (CNTL) with age. Alpha diversity expressed by the Ace (A), Simpson evenness (B), and inverse Simpson (C) indices indicates that richness increases over time, whereas overall diversity and evenness remain more stable over time for virginiamycin- and flavophospholipol-supplemented broilers. Statistical tests were performed using the Kruskal-Wallis test, and *P* values were adjusted for multiple testing using Bonferroni correction. D8, day 8; D28, day 28; D39, day 39. *, *P* < 0.05; **, *P* < 0.01; ***, *P* < 0.001.

To assess the impact of virginiamycin and flavophospholipol supplementations on the ileal flora, alpha diversity was compared with control chickens at each time point (Fig. 3). Virginiamycin-treated chickens were found to have higher richness, which was particularly pronounced at D8 and D39 (*P* = 0.001 and *P* = 0.03, respectively). No significant differences were observed between the flavophospholipol and control groups. The overall alpha diversity and evenness were found to be lower for the virginiamycin group than for flavophospholipol and control chickens (*P* = 0.02 and *P*= 0.04 for D28 evenness and D39 inverse Simpson, respectively). This might indicate that the microbial community became richer but more uneven over time.

**FIG 4:**
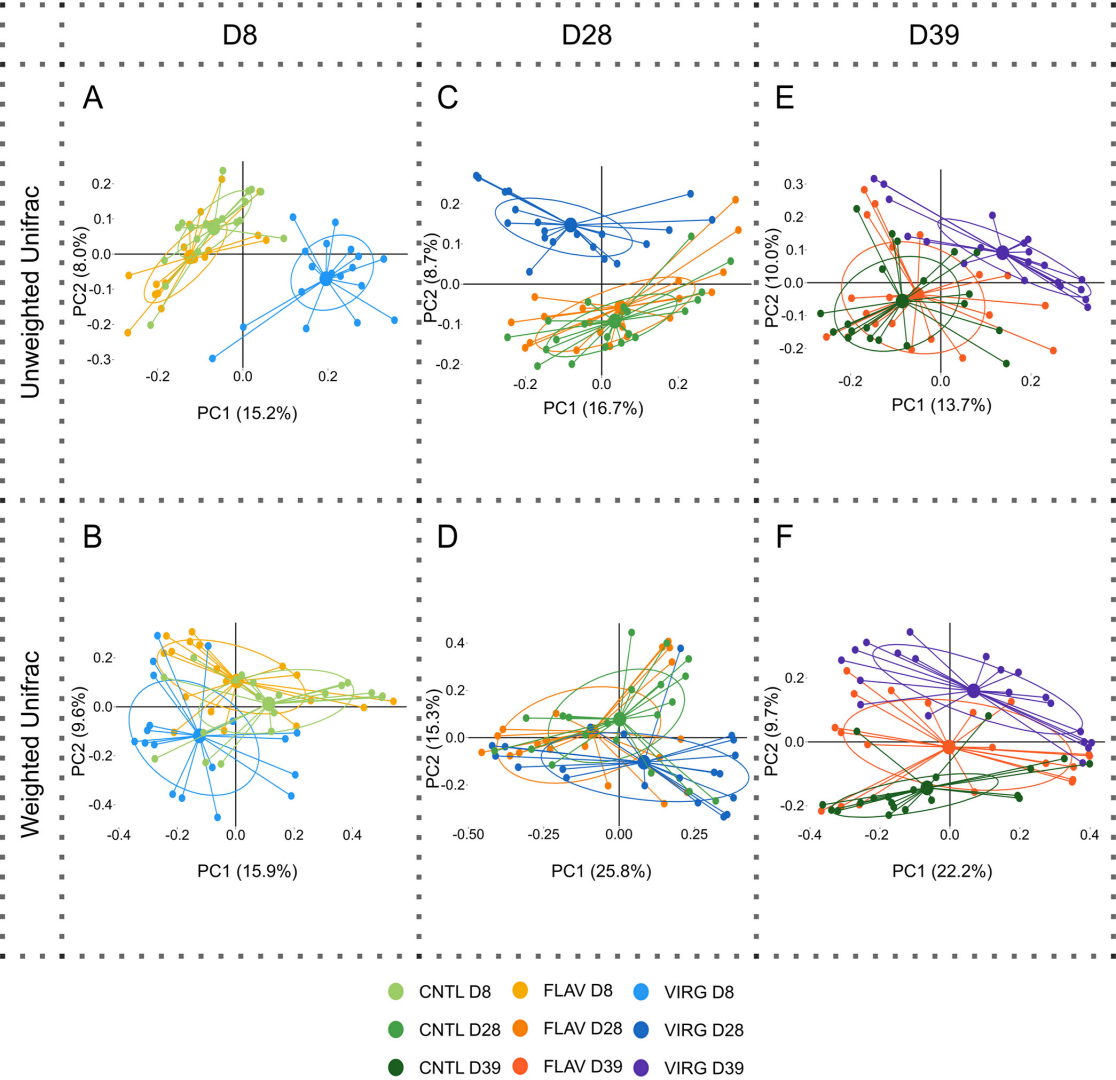
Ileal microbial composition evolves with age in untreated broiler chickens (green) and chickens treated with flavophospholipol (FLAV, 15 ppm, orange) and virginiamycin (VIRG, 20 ppm, blue). Differences in microbial composition were visualized using multidimensional scaling analysis of weighted and unweighted UniFrac distances of samples collected at days 8 (D8), 28 (D28), and 39 (D39). D8 samples are shown in panels A and B; D28 samples in panels C and D; and D39 samples in panels E and F for unweighted and weighted UniFrac, respectively. For detailed information on distinct taxa driving microbial differences reflected in the PCoA, see Table S2 in the supplemental material.

By comparing the beta diversity between groups (analysis of molecular variance [AMOVA] and analysis of similarity [ANOSIM]), it was found that the microbial composition was distinct between the three groups at different time points (Table S5). Specifically, the microbial composition of the virginiamycin group was distinct from both control and flavophospholipol chickens at all time points (*P* ≤ 0.045), whereas the microbial composition of the flavophospholipol and control groups differed only at D39 (*P* < 0.001). Multidimensional scaling analysis similarly showed that control and flavophospholipol-supplemented chickens harbored similar microbial profiles as they clustered together at each of the three time points and were primarily distinguished by lactobacilli and *Enterococcus*, *Blautia*, and Staphylococcus spp. (Fig. 4, Table S2, and Fig. S2). Virginiamycin-supplemented chickens clustered separately from both control and flavophospholipol-supplemented chickens at each time point, although at D39, microbial composition of flavophospholipol-supplemented broilers was more similar to that of virginiamycin-supplemented broilers than to controls (Fig. 4).

10.1128/mSystems.00381-21.2 FIG S2 Ileal microbial composition evolves with age in untreated broiler chickens (green) and chickens treated with flavophospholipol (FLAV, 15 ppm, orange) and virginiamycin (VIRG, 20 ppm, blue). Differences in microbial composition were visualized using multidimensional scaling analysis of weighted and unweighted UniFrac distances of samples collected at days 8 (D8), 28 (D28), and 39 (D39). The three time points are illustrated using unweighted UniFrac in panel A and weighted UniFrac in panel B. Download FIG S2, DOCX file, 0.6 MB. Copyright © 2021 Mysara et al. 2021 Mysara et al. https://creativecommons.org/licenses/by/4.0/ This content is distributed under the terms of the Creative Commons Attribution 4.0 International license.

10.1128/mSystems.00381-21.8 TABLE S5 AMOVA and ANOSIM analysis of control (CNTL), flavophospholipol (FLAV), or virginiamycin (VIRG) broilers at days 8, 28, or 39 (D8, D28, and D39, respectively). Download Table S5, XLSX file, 0.01 MB. Copyright © 2021 Mysara et al. 2021 Mysara et al. https://creativecommons.org/licenses/by/4.0/ This content is distributed under the terms of the Creative Commons Attribution 4.0 International license.

**FIG 5:**
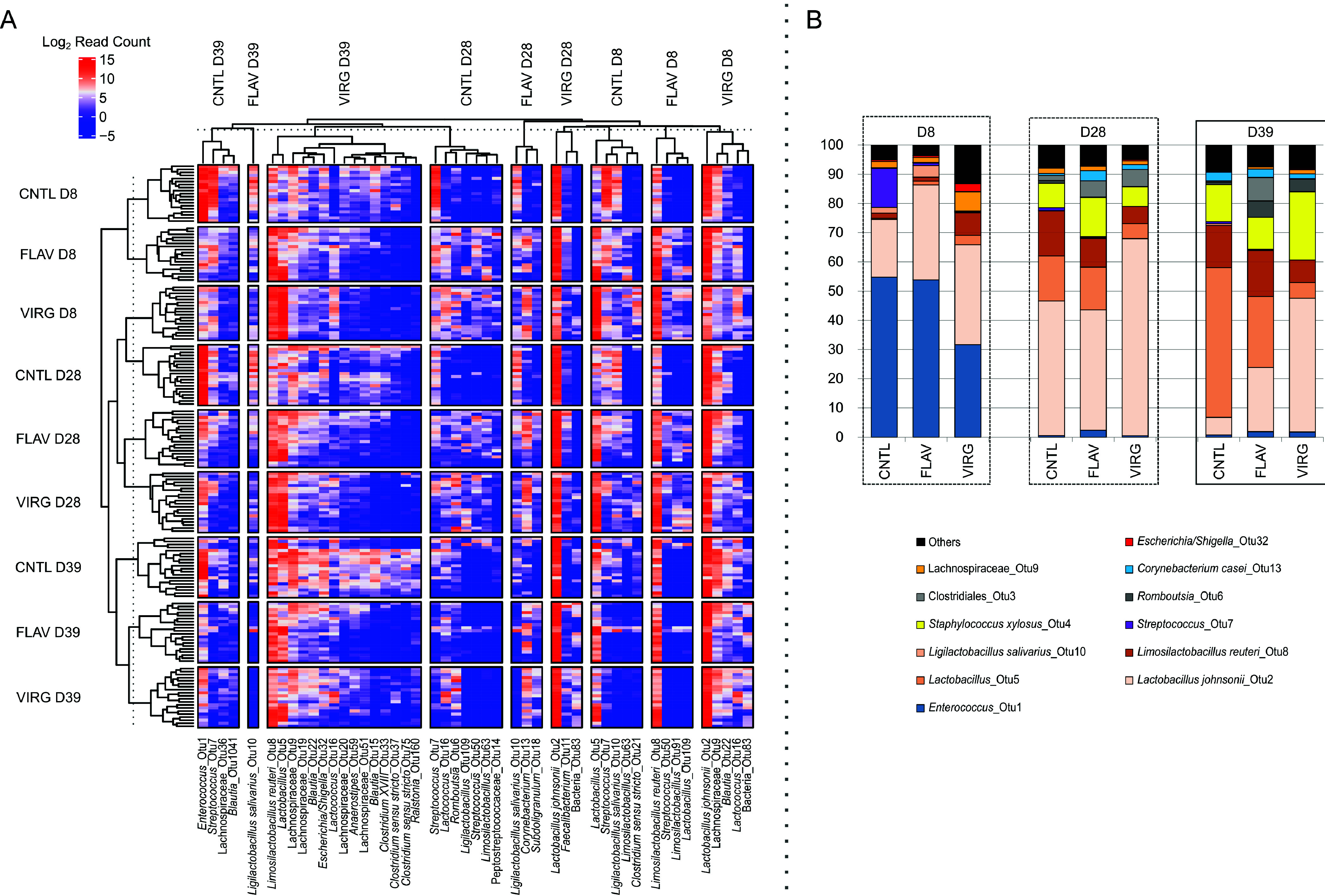
Microbial composition changes with age in flavophospholipol- (FLAV) and virginiamycin-supplemented (VIRG) and control (CNTL) broilers. OTUs differentially abundant between the treatment groups for each time point were identified, and their rarified read counts (log_2_ transformed) were visualized in a heatmap (A). Relative abundances of the most dominant taxa at each time point were further compared between treatment groups using stacked bar plots (B), clearly showing that the composition is dependent on AGP as well as time point. D8, day 8; D28, day 28; D39, day 39.

Differential abundant OTUs were identified across treatment groups at all time points and stratified into distinct types using oligotyping (Table S3). At D8, the control group was found to harbor elevated levels of Streptococcus and *Enterococcus* spp. compared to the flavophospholipol- and virginiamycin-supplemented groups, where the enterococcal abundance was higher in chicks supplemented with flavophospholipol than in those supplemented with virginiamycin (Table 2). Additionally, the virginiamycin group was characterized by a reduced abundance of *L. salivarius* and elevated abundances of members of the *Clostridia* class, such as *Lachnospiraceae* (including *Blautia* spp.), *Clostridium sensu stricto*, and *Clostridium* XVIII. Moreover, Escherichia*/Shigella* and *Corynebacterium* spp., L. crispatus, and L. reuteri were also more abundant in the virginiamycin group than in the flavophospholipol and control groups (Fig. 5 and Fig. S1).

**TABLE 2:** Distinctly altered OTUs (*P* < 0.05) in the ileal broiler microbiota in the control, flavophospholipol, and virginiamycin groups at days 8, 28, and 39 identified by linear discriminant analysis effect size (LDA ≥ 3.0) and Metastats^*a*^

Taxonomy	OTU	Avg abundance (%)			Metastats, *P* value			LEfSe		
		CNTL	FLAV	VIRG	FLAV vs CNTL	VIRG vs CNTL	VIRG vs FLAV	Class	LDA	*P* value
D8										
*Enterococcus*	Otu1	54.8	53.8	31.7	0.93	0.03	0.03	CNTL	5.2	0.036
*Lactobacillus*	Otu5	0.3	1.3	3.1	0.34	0	0.41	VIRG	4.2	0.003
Streptococcus	Otu7	13.4	0.9	0.3	0	0	0.11	CNTL	4.8	0
*Limosilactobacillus*	Otu8	1.8	1.4	7.7	0.66	0.03	0.03	VIRG	4.7	0.017
*Lachnospiraceae*	Otu9	2.1	1.7	6.6	0.8	0.01	0	VIRG	4.4	0.001
*Ligilactobacillus*	Otu10	2	4	0.1	0.6	0	0	FLAV	4.2	0
*Blautia*	Otu15	1	0.3	1.5	0.12	0.5	0	VIRG	3.8	0.004
*Lachnospiraceae*	Otu19	0.7	0.3	1.9	0.21	0.07	0	VIRG	3.9	0.028
*Lactococcus*	Otu16	0	0	0.3	0.2	0	0	VIRG	3.1	0
*Lachnospiraceae*	Otu20	0.1	0.2	0.4	0.85	0.01	0.05	VIRG	3.1	0.004
*Blautia*	Otu22	0	0.1	0.7	0.13	0	0	VIRG	3.6	0
Escherichia/*Shigella*	Otu32	0.6	0.7	2.7	0.7	0	0.01	VIRG	3.9	0
*Clostridium* XVIII	Otu33	0.1	0.1	0.3	0.7	0.06	0.02	VIRG	3.1	0.002
*Lachnospiraceae*	Otu36	0.2	0	0.2	0.16	0.93	0.01	CNTL	3	0.033
*Clostridium sensu stricto*	Otu37	0.1	0	0.4	0.24	0.51	0.01	VIRG	3.1	0.01
*Lachnospiraceae*	Otu51	0.1	0.1	0.6	0.57	0	0	VIRG	3.5	0
*Anaerostipes*	Otu59	0.2	0	0.4	0.26	0.23	0	VIRG	3.2	0.001
*Blautia*	Otu1041	0.2	0.1	0	0.23	0.05	0.13	CNTL	3.2	0.014
*Clostridium sensu stricto*	Otu75	0	0	0.5	0.01	0	0	VIRG	3.5	0
*Ralstonia*	Otu160	0	0	0.2	1	0	0	VIRG	3	0

D28										
*Lactobacillus*	Otu2	46.1	41.2	67.5	0.5	0.01	0	VIRG	5	0.003
*Romboutsia*	Otu6	0.9	0.1	0	0.68	0.12	0.01	CNTL	3.6	0.032
Streptococcus	Otu7	1	0.3	0	0.07	0	0	CNTL	3.7	0
*Faecalibacterium*	Otu11	0.6	0.7	0.7	0.92	0.97	0.99	VIRG	3.9	0.001
*Ligilactobacillus*	Otu10	0.1	0.3	0	0.29	0	0	FLAV	3.1	0
*Corynebacterium*	Otu13	0.7	3.5	1.7	0.01	0.21	0.16	FLAV	4.2	0.034
*Peptostreptococcaceae*	Otu14	1.5	0.1	0	0.79	0	0	CNTL	3.9	0
*Subdoligranulum*	Otu18	0	0.3	0.1	0.16	0.49	0.49	FLAV	3.2	0.048
*Lactococcus*	Otu16	0.8	0.1	0.2	0	0.21	0.31	CNTL	3.5	0.001
Streptococcus	Otu50	0.5	0.4	0	0.76	0	0	CNTL	3.4	0
*Limosilactobacillus*	Otu63	0.7	0.1	0	0.03	0	0	CNTL	3.5	0
*Bacteria*	Otu83	0	0	0.5	1	0	0	VIRG	3.5	0
*Ligilactobacillus*	Otu109	0.4	0.2	0	0.52	0	0	CNTL	3.3	0

D39										
*Lactobacillus*	Otu2	6.1	22	45.8	0	0	0	VIRG	5.3	0
*Lactobacillus*	Otu5	51.2	24.2	5.4	0	0	0	CNTL	5.4	0
Streptococcus	Otu7	0.7	0.3	0	0.15	0	0.04	CNTL	3.6	0
*Limosilactobacillus*	Otu8	14.4	15.7	7.6	0.7	0.02	0.02	FLAV	4.7	0.008
*Lachnospiraceae*	Otu9	0.2	0.7	1.4	0.25	0	0.21	VIRG	3.8	0.018
*Ligilactobacillus*	Otu10	0.5	0.2	0	0.47	0	0	CNTL	3.5	0
*Lactococcus*	Otu16	1.1	0.3	1.4	0.01	0.73	0.16	VIRG	3.8	0.003
*Clostridium sensu stricto*	Otu21	1.5	0.3	0	0.02	0	0	CNTL	3.8	0
*Blautia*	Otu22	0.1	0.2	0.4	0.28	0	0.04	VIRG	3.2	0.006
Streptococcus	Otu50	0.1	0.3	0	0.19	0	0	FLAV	3.1	0
*Limosilactobacillus*	Otu63	0.2	0	0	0.48	0	0	CNTL	3	0
*Bacteria*	Otu83	0.1	0	0.3	0.4	0	0	VIRG	3.1	0
*Limosilactobacillus*	Otu91	0.2	0.3	0	0.86	0	0	FLAV	3.2	0
*Ligilactobacillus*	Otu109	0.3	0.3	0	0.72	0	0	FLAV	3.2	0

a Abbreviations: CNTL, control; FLAV, flavophospholipol; VIRG, virginiamycin; LDA, linear discriminant analysis; LEfSe, LDA effect size; D8, day 8; D28, day 28; D39, day 39.

At D28, virginiamycin group samples were characterized by elevated levels of L. johnsonii and *Faecalibacterium* spp. (Otu11), whereas the flavophospholipol group was characterized by elevated levels of *Corynebacterium* spp. and *L. salivarius*, and the control group by taxa belonging to the *Streptococcaceae* and *Peptostreptococcaceae* families (including, e.g., *Romboutsia* spp.).

**FIG 6:**
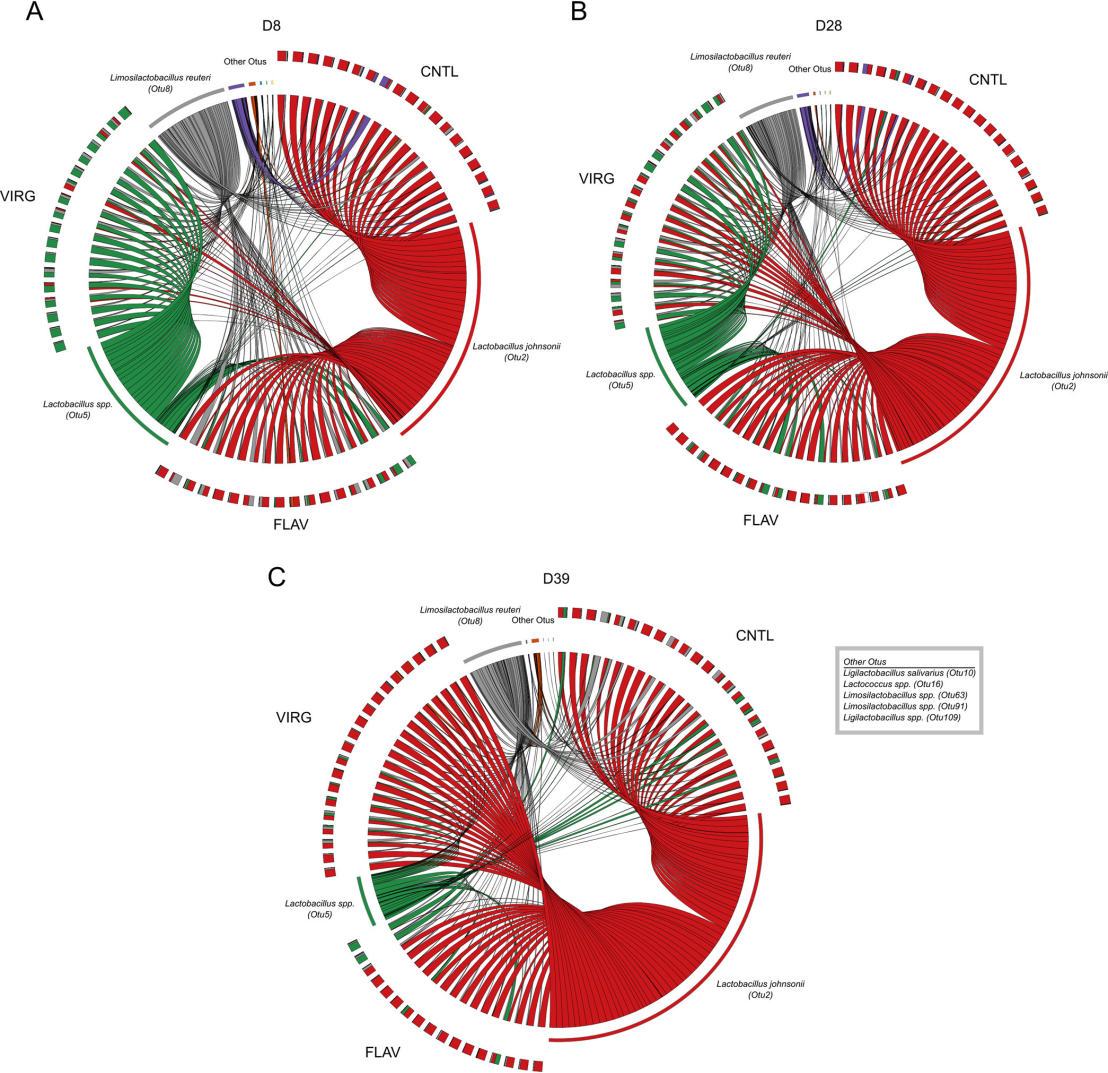
*Lactobacillus* dynamics in the ileal microbiota in the broiler chicken are both age and AGP dependent. Relative abundances of members of the *Lactobacillus* community in the control (CNTL), flavophospholipol (FLAV), and virginiamycin (VIRG) groups at days 8 (D8) (A), 28 (D28) (B), and 39 (D39) (C) were visualized using Circos, indicating the shifting dynamics of the species harbored within.

Ultimately, at D39, L. crispatus/*L. gallinarum* and *L. salivarius* as well as *S. pasteurianus*/*S. infantarius*/*S. alactolyticus*/*S*. *macedonicus* had distinctly higher relative abundance in the control group than in the flavophospholipol and virginiamycin groups. Flavophospholipol-treated chickens harbored elevated levels of L. reuteri and Streptococcus spp., while *Lachnospiraceae* members (including *Blautia* spp.), and L. johnsonii were more abundant in the virginiamycin group than in the other two (Fig. 5). The differences in composition of the most dominant members among the lactobacilli across each treatment group at the three time points are illustrated in Fig. 6.

### AGP supplementation influences metabolic activity at D8 and D39.

Functional assessment of the microbial changes was performed for both the virginiamycin and flavophospholipol groups at each time point, which revealed differences between the AGP-supplemented chickens and the control group at D8 and D39 for virginiamycin, whereas no differences were observed at D28 (Table S6). At D8, both virginiamycin- and flavophospholipol-supplemented chickens harbored microbiotas with lower galactose degradation and secondary metabolite biosynthesis, driven mainly by the higher abundance of Streptococcus spp. Additional differences in metabolic activity observed between AGP groups were unique to each group.

10.1128/mSystems.00381-21.9 TABLE S6 PICRUSt analysis showing distinctly altered pathways in the ileal broiler microbiota in the control (CNTL), flavophospholipol (FLAV), and virginiamycin (VIRG) groups at days 8, 28, and 39 (D8, D28, and D39, respectively). Download Table S6, XLSX file, 0.04 MB. Copyright © 2021 Mysara et al. 2021 Mysara et al. https://creativecommons.org/licenses/by/4.0/ This content is distributed under the terms of the Creative Commons Attribution 4.0 International license.

The microbiota within the flavophospholipol group had substantially lower activity in pathways linked to glycan and starch degradation, mainly due to lower abundances of Streptococcus spp., as well as ergothioneine biosynthesis at D8, whereas the control group was more characterized by activity in pathways linked to folic acid, vitamin, and amino acid biosynthesis. Virginiamycin-supplemented chickens, on the other hand, harbored a microbiota associated with elevated metabolic activity linked to biosynthesis of amino acids (particularly tryptophan as a result of elevated levels of enterococcal and *Lachnospiraceae* spp.), ubiquinol/menaquinol (linked to L. johnsonii), and fatty acids (particularly mycolate and palmitoleate, linked to higher abundances of L. johnsonii, L. reuteri, and *Lachnospiraceae* spp.) at D8. Additionally, nucleoside (particularly adenosine and guanosine) and sugar (particularly xylose and glucose) degradation was potentially more common in the virginiamycin-supplemented group at D8 than in the other groups also due to the observed higher abundances of L. johnsonii, L. reuteri, and *Lachnospiraceae* spp.

At D39, distinct differences in metabolic activity were observed only between the control and virginiamycin groups, whereas flavophospholipol-supplemented animals harbored metabolic activity resembling both the control and virginiamycin groups with smaller intermediate changes. The virginiamycin-supplemented group was characterized by higher activity in pathways linked to pentose phosphate (mainly linked to L. johnsonii), biosynthesis of menaquinol (linked to L. johnsonii and L. reuteri), and degradation of the sugars d-galacturonate (linked to L. reuteri and *Lachnospiraceae* spp.) and fructose and rhamnose (linked to *Enterococcus* spp.), and uronic acids (particularly hexuronate and galacturonate, linked to *Enterococcus* spp.). In relation to the virginiamycin-supplemented animal group, the control group was characterized by biosynthesis of amino acids (particularly lysine and threonine), unsaturated fatty acids (particularly vaccinate and gondoate), and degradation of sucrose as a result of elevated abundances of L. crispatus/*L. gallinarum*.

## DISCUSSION

The broiler chicken gut microbiota plays an essential role in animal health and development. The use of AGPs is common in order to enhance growth, but their effect on the gut microbiota and the resulting impact on metabolic activity are largely unknown. Therefore, this controlled intervention study aimed to assess the effect of two commonly used AGPs, namely, virginiamycin (20 ppm) and flavophospholipol (15 ppm), on the broiler chicken microbiota over a period of 6 weeks. Our preliminary results suggested that the AGP-induced alterations were more substantial in the ileum and were consistent with earlier studies showing the proximal gut microbiota to be more susceptible to antibiotics than the distal gut (27, 28). Therefore, we focused on the ileum microbiota in this study.

By comparing microbial compositions in longitudinally collected samples from the broiler chicken ileum at three distinct time points (D8, D28, and D39), we showed that the ileal microbiota at D8 was primarily dominated by enterococci, lactobacilli, and Streptococcus spp., accounting for approximately 90% of the overall community. Lactobacilli (formerly collectively classified as members within the *Lactobacillus* genus) were largely composed of Lactobacillus johnsonii, Lactobacillus crispatus/Lactobacillus gallinarum, Limosilactobacillus reuteri, and Ligilactobacillus salivarius and corroborated previous studies describing the ileal microbiota within the first week of life (29). With age (at D28 and D39), the relative abundance of *Enterococcus* spp. decreased and the abundance of lactobacilli increased, the latter constituting approximately 70% of the microbial community. At these ages, an increased dominance of lactobacilli, primarily of L. johnsonii, L. crispatus/*L. gallinarum*, and L. reuteri was observed, whereas the *L. salivarius* relative abundance was reduced compared to that at D8, in accordance with previous findings (9, 30, 31). We observed further rearrangements within the *Lactobacillus* community between D28 and D39 (mainly from L. johnsonii to L. crispatus/*L. gallinarum*). This dynamic shift in lactobacilli has also been observed in the chicken ileum microbiota at day 42 compared to day 21 (31). Lactobacilli overall enhance protease trypsin and lipase enzymatic activity (32), where *L. salivarius* additionally has the ability to stimulate butyrate-producing bacteria and to reestablish the balance of the microbiota (33). It has been hypothesized previously that this might be attributed to the spatial environment maturation including pH, osmolarity, atmosphere, and availability of bacterial substrates (34). Thus, this genus plays an essential role in regulating starch metabolism and lactate fermentation and also in providing colonization resistance against pathogens, such as Campylobacter jejuni, Campylobacter coli, Salmonella enterica, Escherichia coli, and C. perfringens (35).

When investigating the impact of the two AGPs on the ileal microbiota, we found an AGP-specific effect on both microbial composition and metabolic activity which persisted over time. Both AGPs exhibit activity against Gram-positive bacteria (5, 36), yet the observed effect of virginiamycin on Gram-positive bacteria was more disruptive in this study. This was exemplified by a reduction of Gram-positive bacteria such as Streptococcus spp. and members of the *Peptostreptococcaceae* family. Virginiamycin further affected the relative abundances of *Faecalibacterium* spp. and *Enterococcus* spp., whereas they remained unaffected by flavophospholipol. Additional taxa, primarily *Clostridiales* members such as *Blautia* spp., *Lachnospiraceae* spp., *Clostridium sensu stricto* spp., and *Clostridium* XVIII spp., were found to increase drastically after virginiamycin exposure, whereas flavophospholipol treatment resulted in no or minor changes. Members of the *Clostridiales* order, particularly *Lachnospiraceae* spp., are producers of SCFAs such as formic and butyric acid. These SCFAs play an important role in cellulose and starch degradation and provide colonization resistance against pathogens in poultry (18), which partially explains the mode of action of virginiamycin as a growth promoter.

In this study, AGP exposure resulted in adaptations of lactobacilli characterized by a dynamic shift between the different species within this taxon, driven by competitive exclusion as also observed previously (28). Lactobacilli facilitate uptake of the SCFA butyrate, produced by other bacteria, by intestinal epithelial cells (37). Butyrate maintains intestinal epithelial integrity, ameliorates mucosal inflammation, and stimulates electrolyte (NaCl) absorption, playing an important role in gut health (38, 39), and its impairment could result in intestinal inflammatory diseases. Further, butyrate is involved in regulation of inflammation, cell differentiation, and apoptosis in the host. In this study, virginiamycin-supplemented chickens were characterized by a higher abundance of L. johnsonii and a depletion of other lactobacilli, namely, L. crispatus/*L. gallinarum*, L. reuteri, and *L. salivarius.* For flavophospholipol, such a disruption in lactobacilli was reported only at D39, with high levels of L. johnsonii and a depletion of L. crispatus and *L. gallinarum*. However, in contrast to virginiamycin-supplemented broilers, no distinct alterations in L. reuteri or *L. salivarius* abundances were found. *L. salivarius* is a probiotic bacterium associated with a microbiota with increased butyrate production after supplementation, indicating its role in a healthy microbial flora (40, 41). This enrichment of L. johnsonii after virginiamycin exposure might indicate an intrinsic resistance to protein synthesis inhibitors, in contrast to several other lactobacilli (42, 43).

Although early hypotheses suggested that AGPs exhibit their effect by diminishing microbial presence in the gut and thus rendering more nutrients available for the host (44), recent theories suggest more complex underlying mechanisms. AGPs are likely to promote a balanced microbiota less likely to trigger immune and inflammatory responses, thereby inhibiting subclinical infections and increasing the bioavailability of nutrients (45). In this study (45), both the flavophospholipol- and virginiamycin-supplemented microbiota displayed reduced galactose degradation compared to the control. Previous studies have reported an increased weight gain as a result of larger amounts of galactose in food (46), indicating a possibly increased bioavailability of galactose as a result of AGP supplementation. Furthermore, flavophospholipol-supplemented broilers displayed higher ergothioneine biosynthesis compared to the other groups at D8. This amino acid is not naturally synthesized by animals and is believed to play a role in free radical scavenging and inflammatory modulation after being taken up by the animal tissue (47). Additionally, the flavophospholipol-supplemented broiler microbiota showed a potentially reduced starch and glycan degradation compared to other groups, indicating a possible increase in bioavailability for the host.

Virginiamycin-supplemented broilers similarly showed possible increases in nutrient bioavailability after supplementation. Metabolism of amino acids (primarily tryptophan), fatty acid biosynthesis, and sugar and carbohydrate degradation in virginiamycin-supplemented broilers were higher, supporting a previous report (45). This study argues that increased levels of tryptophan are accompanied by increased kynurenine, reduction in serotonin, and a possible decrease in ileum movement that allows prolonged nutrient absorption as a result (45). Additionally, ubiquinol-enhanced biosynthesis in the virginiamycin-treated broilers may play an anti-inflammatory role (48), allowing for a better environment for nutrient intake. Finally, at D39, virginiamycin-supplemented broilers displayed increased activity in the pentose phosphate pathway (PPP), where PPP metabolite-elevated flux is suggested to facilitate muscle growth and regeneration in chickens (49).

While both virginiamycin and flavophospholipol resulted in altered metabolic activity, possibly increasing the bioavailability of essential nutrients, virginiamycin-supplemented broilers also displayed an increased relative abundance of opportunistic pathogens. At D8, virginiamycin-supplemented broilers harbored elevated levels of Clostridium perfringens similar to what has been reported previously and with a suggested impact on mortality (15). Furthermore, virginiamycin-supplemented broilers harbored elevated levels of Campylobacter and Escherichia*/Shigella* spp. at D8 and D28. Previous reports indicate that this contributes to the intrinsic resistance of E. coli to virginiamycin, which could explain their increase as a result of the elimination of other sensitive bacteria. Although E. coli can naturally exist in low abundances in the chicken microbiota (34), certain strains can cause disease when exhibiting specific virulence traits of avian pathogenicity (50).

In this controlled intervention study, we have utilized a virginiamycin dose similar to those used in food animal farming whereas the flavophospholipol dose was slightly higher. Although the trial design is similar to the regular farming environment, the experimental design does not mimic the exact condition seen in food production where stress is induced by housing of thousands of animals. Setting up a controlled study under such conditions is costly and thereby not feasible for such a detailed investigation into the ileal microbiota of the broiler chicken. Thus, it was not possible to capture the actual stress levels the animals might be exposed to resulting in higher loads of pathogens than observed here as well as increased mortality rates.

This study provides a first head-to-head comparison of the effect of the two AGPs flavophospholipol and virginiamycin on the broiler chicken ileum microbiota over time. Here, virginiamycin was revealed to have an immediate and prolonged effect on microbial composition and metabolic activity in the broiler ileum persisting until D39. Although an altered microbial composition was observed in flavophospholipol-supplemented broilers at D39, the observed differences were milder than in those exposed to virginiamycin. Both AGPs resulted in metabolic changes potentially resulting in increased anti-inflammatory mechanisms as well as nutrient bioavailability of several essential nutrients, either by decreasing their degradation (primarily after flavophospholipol supplementation) or by increasing their biosynthesis (primarily after virginiamycin supplementation). A head-to-head comparison indicates a small benefit in using flavophospholipol over virginiamycin as the two have been shown to result in similar weight gains, whereas virginiamycin results in more extensive perturbations in ileal microbial composition and increased colonization with potentially pathogenic bacteria such as Campylobacter spp., Clostridium perfringens, and Escherichia coli, which could impact broiler mortality.

## MATERIALS AND METHODS

### Experimental design and sample collection.

A 39-day controlled intervention study assessing the impact on the ileal and cecal microbiota of the broiler chicken was performed in Bocholt, Belgium, during 2015. A total of 180 male broiler chickens purchased from a commercial vendor were randomly divided into three groups and studied for a period of 6 weeks. The animals were randomly divided over 30 pens in the same room. The different groups were fed *ad libitum* with starter feed from day 1 to 14 and grower and finished feed from day 15 to 39 supplemented with either flavophospholipol (15 ppm), virginiamycin (20 ppm), or control (without APG supplementation); see Fig. S3 in the supplemental material.

10.1128/mSystems.00381-21.3 FIG S3 Rarefaction curves of sequenced samples within the study. Download FIG S3, DOCX file, 0.2 MB. Copyright © 2021 Mysara et al. 2021 Mysara et al. https://creativecommons.org/licenses/by/4.0/ This content is distributed under the terms of the Creative Commons Attribution 4.0 International license.

Twenty chickens per supplementation group were sacrificed by a licensed veterinarian via cervical dislocation followed by dissection at three defined time points, day 8 (D8), day 28 (D28), and day 39 (D39), where ileal and cecal samples were collected into stool collection tubes (Sarstedt; catalog no. 80.734.001). One chicken died in the control group prior to D8, one in the flavophospholipol-supplemented group prior to D28, and two in the virginiamycin-supplemented group prior to D8. An additional two samples were lost in the flavophospholipol-supplemented group at D8 due to failure of collection or insufficient sample material. Collected samples were refrigerated until transportation to the Laboratory of Medical Microbiology (LMM), University of Antwerp, could be arranged. Transportation was arranged on the same day as samples were collected at each time point and stored at −80°C until further processing. After a pilot analysis of the collected samples, it was decided to proceed only with the ileum samples as these were found to be more sensitive to the effect of the investigated AGPs (data not shown).

### DNA extraction and quality control.

Samples were thawed in batches, and 500 mg ileal or cecal content was weighed and added to a 2-ml lysing matrix E tube to extract the total metagenomic DNA using the FastDNA spin kit (MP Biomedicals, Irvine, CA) according to the manufacturer’s instructions. An additional purification step was performed with the DNA Clean and Concentrator kit (Zymo Research, Irvine, CA, USA) before the DNA concentration was determined with a Qubit 2.0 fluorometer utilizing the double-stranded DNA high-sensitivity assay kit (Thermo Fisher Scientific).

### 16S rRNA gene amplification, library preparation, and sequencing.

PCR amplification of the V3-V4 regions of the 16S rRNA gene was performed using standard Illumina fusion primers (341F and 802R) with 2× Kapa HiFi Hot Start Ready mix (Kapa Biosystems) as master mix using the following cycling parameters: initial denaturation at 95°C for 3 min; 25 cycles of 95°C for 30 s, 60°C for 30 s, and 72°C for 30 s; and elongation at 72°C for 10 min in triplicates of each sample. Multiplexed libraries were prepared with the Nextera XT kit (Illumina Inc., USA) and sequenced using MiSeq V2 chemistry with 500 cycles with 2 × 250-bp paired-end sequencing. The sequencing data were processed and demultiplexed using Illumina CASAVA (1.8.2), and adapters were trimmed with skewer v.0.1.116.

### Data analysis.

The 16S rRNA amplicon sequencing data preprocessing was conducted using the OCToPUS pipeline (51). In summary, contigs were created by heuristically merging paired-end reads in mothur v1.39 (52) based on the Phred quality score of both reads. Contigs were aligned to the SILVA v.132 database (53) and filtered from (i) those with ambiguous bases, (ii) those with more than 8 homopolymers, (iii) those with a length below 390, and (iv) those not corresponding to the V3-V4 region. The aligned sequences were filtered and dereplicated while sequencing errors were removed using the IPED v1.0 algorithm, which is dedicated to denoising MiSeq amplicon sequencing data (54). Chimera removal was performed with our in-house-developed tool called CATCh v1.0 (55) in *de novo* mode. Sequences were clustered into operational taxonomic units (OTUs) with the USEARCH v8.1.186 implementation of UPARSE (56) using the default settings. Taxonomic classification was performed using the RDP database v.18 (57). Rarefaction curves were constructed to assess saturation, and samples were rarefied to the smallest sample size to homogenize the sequencing depth allowing proper statistical comparison (Fig. S3).

Various alpha diversity indices, such as Ace, inverse Simpson, and Simpson evenness, together with the weighted and unweighted UniFrac beta diversity distances, were calculated in mothur. Statistical comparison of alpha diversity indices was conducted using either parametric analysis of variance (ANOVA) or the nonparametric Kruskal-Wallis test, and the *P* values were corrected for multiple testing using Bonferroni correction. Parametric testing was applied when both the normality (Shapiro’s test, using R *shapiro.test*) and homoscedasticity (Bartlett’s test, using R *bartlett.test*) were confirmed; otherwise, nonparametric testing was applied. Beta diversity distances were analyzed using multidimensional scaling by constructing principal-coordinate analysis (PCoA) plots, and OTUs were correlated with the coordinates using the *corr.axes* command in mothur. Hypothesis testing was performed using AMOVA and ANOSIM (58), using weighted and unweighted UniFrac distances. Differentially abundant OTUs were assessed by linear discriminant analysis effect size (LEfSe [59]), and these identified OTUs were further assessed using *metastats* (60), which utilizes a nonparametric *t* test-like approach.

Further stratification of OTUs was conducted for OTUs of interest using oligotyping (61). Oligotyping can distinguish down to single-nucleotide differences in 16S rRNA gene sequences while disregarding sequencing errors. Identified oligotypes were further classified using NCBI nucleotide BLAST where sequences were classified as the best hit exceeding an identity of 97%. Functional analysis was conducted with PICRUSt2 v.2.1.3 (62) with the MetaCyc database v.24.5 (63) as reference where statistical comparisons between groups were conducted in STAMP v.2.1.3 (64). Pathway comparisons were performed using nonparametric testing and false-discovery rate (FDR) correction of *P* values. The pathways with *P* values of <0.05 and with a ratio of proportions exceeding 2 were reported.

### Ethical approval.

All study-related activities in this project were conducted within the ethical regulations and standards set and carried out by the PVL (Agricultural Testing and Training Center, North Limburg, Belgium).

### Data availability.

Sequence data generated in this study have been made available at the Sequence Read Archive (SRA) on NCBI under project number PRJNA679035.
