# Supplementary figures and images for: Assessing the Impact of Flavophospholipol and Virginiamycin Supplementation on the Broiler Microbiota: a Prospective Controlled Intervention Study
Source: mSystems. 2021 Aug 31;6(4):10.1128/msystems.00381-21. doi: 10.1128/msystems.00381-21 (PMC12338151; doi:10.1128/msystems.00381-21)

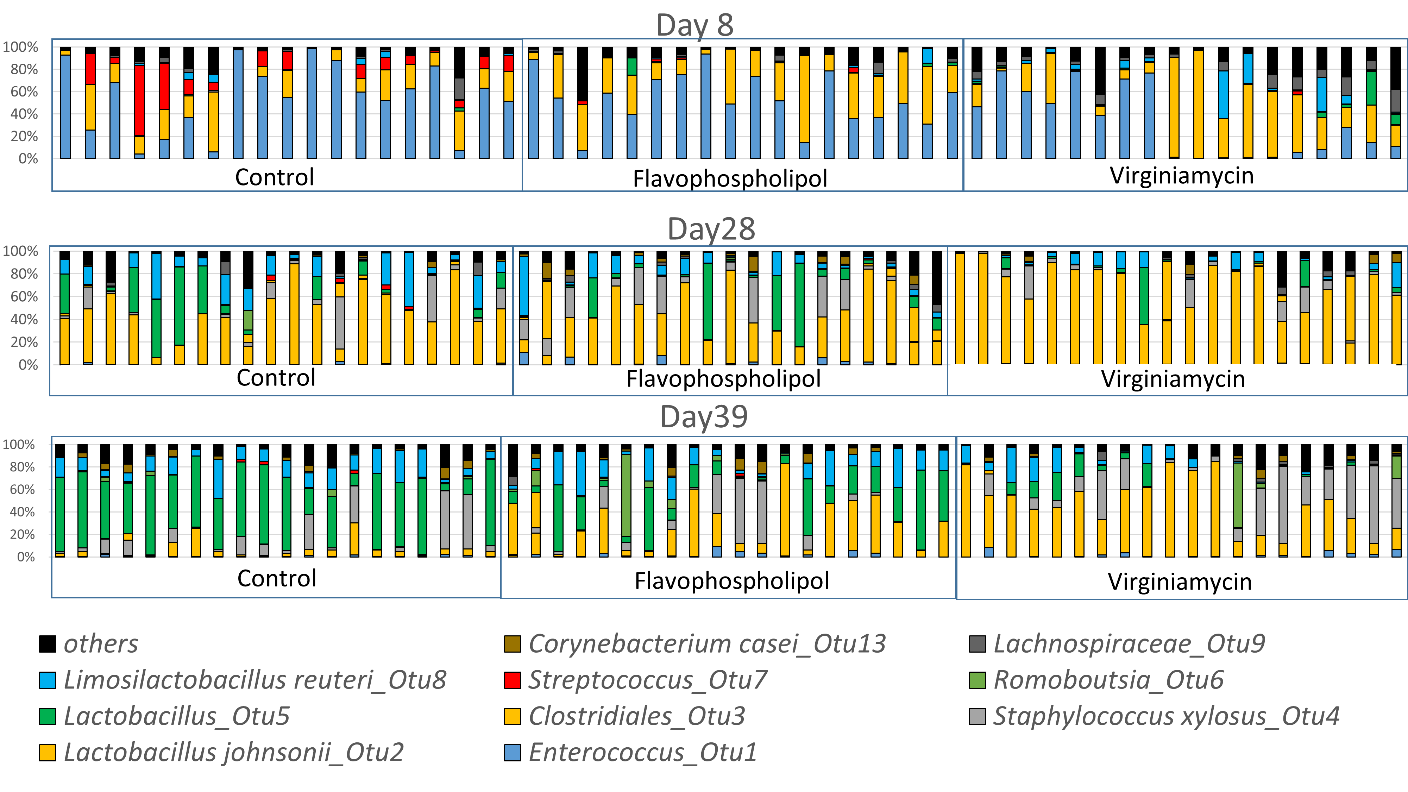

Supplement: FIG S1 [file msystems.00381-21-sf001.docx]

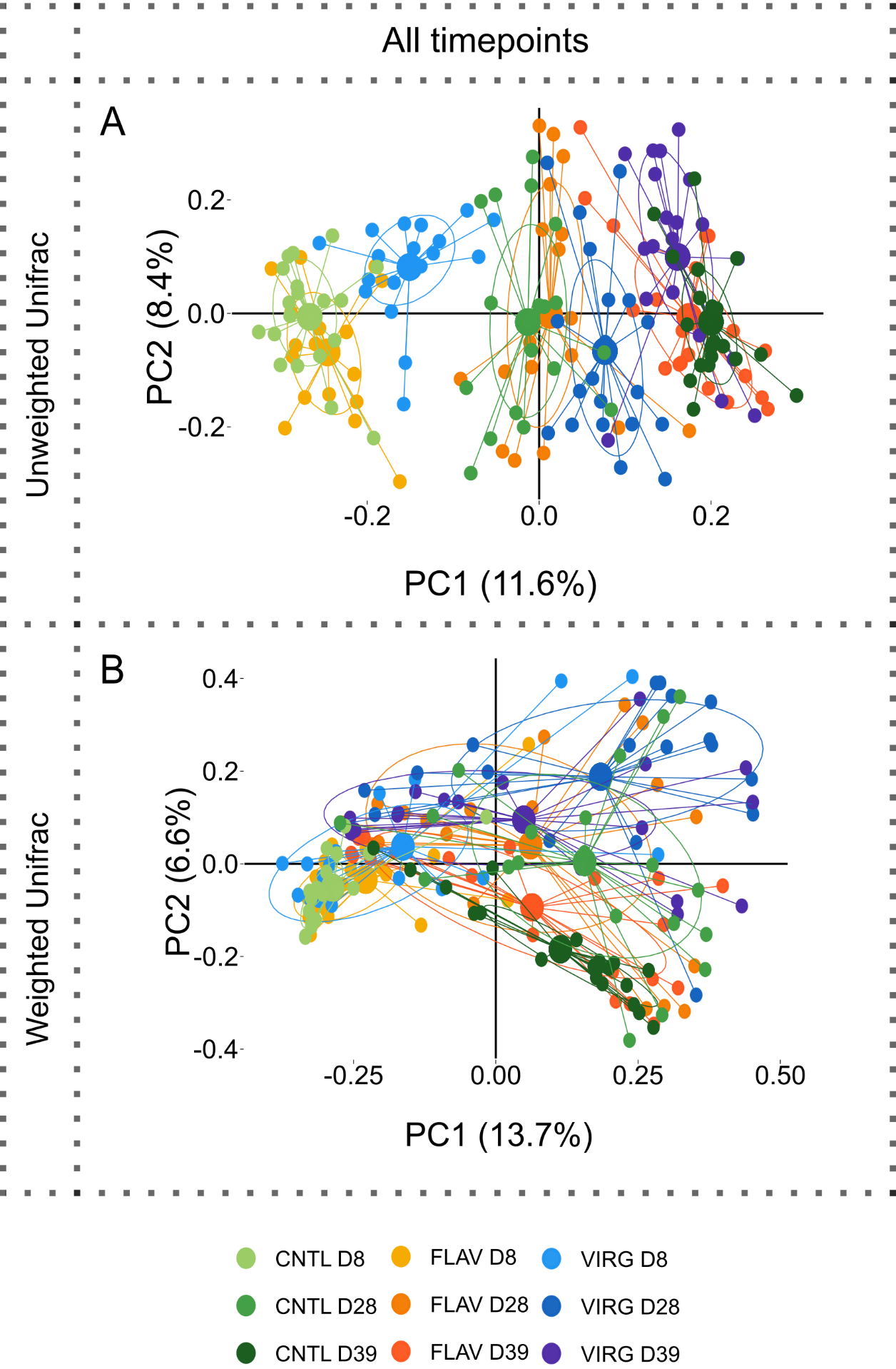

Supplement: FIG S2 [file msystems.00381-21-sf002.docx]

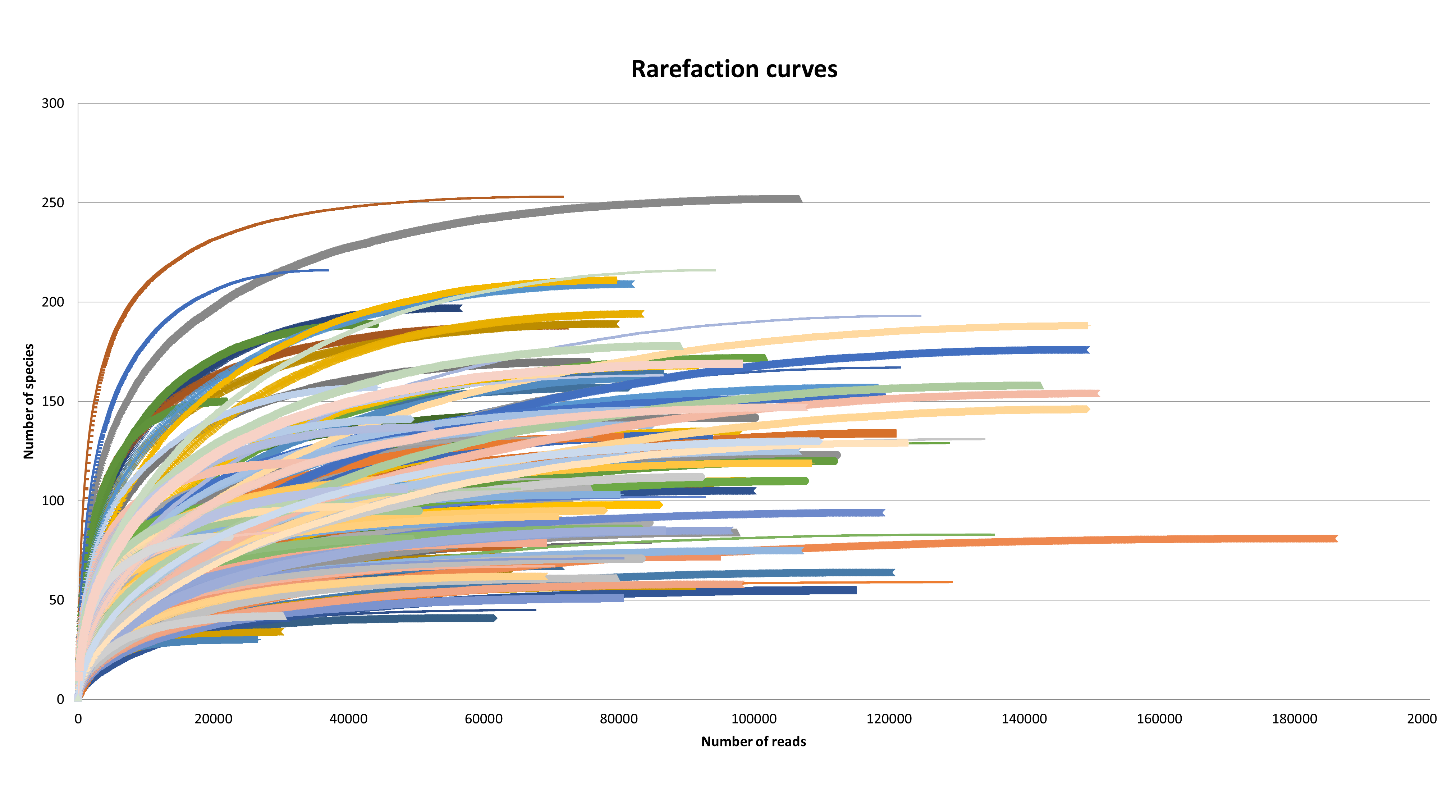

Supplement: FIG S3 [file msystems.00381-21-sf003.docx]
